# Biological and Mechanical Performance of Dual-Setting Brushite–Silica Gel Cements

**DOI:** 10.3390/jfb15040108

**Published:** 2024-04-18

**Authors:** Valentin C. Steinacker, Tobias Renner, Ib Holzmeister, Sebastian Gubik, Urs Müller-Richter, Niko Breitenbücher, Andreas Fuchs, Anton Straub, Mario Scheurer, Alexander C. Kübler, Uwe Gbureck

**Affiliations:** 1 Department of Oral & Maxillofacial Plastic Surgery, University Hospital Würzburg, Pleicherwall 2, 97070 Würzburg, Germany; 2 Department for Functional Materials in Medicine and Dentistry, University Hospital Würzburg, Pleicherwall 2, 97070 Würzburg, Germany; 3 Department of Cranio-Maxillo-Facial-Surgery, German Armed Forces Hospital Ulm, 89081 Ulm, Germany

**Keywords:** brushite, dual-setting, silica gel, biocompatibility

## Abstract

Bone defects resulting from trauma, diseases, or surgical procedures pose significant challenges in the field of oral and maxillofacial surgery. The development of effective bone substitute materials that promote bone healing and regeneration is crucial for successful clinical outcomes. Calcium phosphate cements (CPCs) have emerged as promising candidates for bone replacement due to their biocompatibility, bioactivity, and ability to integrate with host tissues. However, there is a continuous demand for further improvements in the mechanical properties, biodegradability, and bioactivity of these materials. Dual setting of cements is one way to improve the performance of CPCs. Therefore, silicate matrices can be incorporated in these cements. Silicate-based materials have shown great potential in various biomedical applications, including tissue engineering and drug delivery systems. In the context of bone regeneration, silicate matrices offer unique advantages such as improved mechanical stability, controlled release of bioactive ions, and enhanced cellular responses. Comprehensive assessments of both the material properties and biological responses of our samples were conducted. Cytocompatibility was assessed through in vitro testing using osteoblastic (MG-63) and osteoclastic (RAW 264.7) cell lines. Cell activity on the surfaces was quantified, and scanning electron microscopy (SEM) was employed to capture images of the RAW cells. In our study, incorporation of tetraethyl orthosilicate (TEOS) in dual-curing cements significantly enhanced physical properties, attributed to increased crosslinking density and reduced pore size. Higher alkoxysilyl group concentration improved biocompatibility by facilitating greater crosslinking. Additionally, our findings suggest citrate’s potential as an alternative retarder due to its positive interaction with the silicate matrix, offering insights for future dental material research. This paper aims to provide an overview of the importance of silicate matrices as modifiers for calcium phosphate cements, focusing on their impact on the mechanical properties, setting behaviour, and biocompatibility of the resulting composites.

## 1. Introduction

Calcium phosphate cements have gained considerable attention as bone substitutes due to their resemblance to the mineral phase of natural bone, their ability to support bone regeneration, and their potential for integration with host tissues.

Hydroxyapatite is the most stable calcium phosphate cement with compressive strength ranging up to 180 MPa [1,2] and tensile strength ranging from 10–16 MPa [3].

Brushite has a significantly lower compressive strength of up to 60 MPa and tensile strength of up to 10 MPa, making it suitable for use in less load-bearing areas, such as the face, as a bone substitute material [4]. However, the setting reaction of this cement is much faster than that of hydroxyapatite cements, taking only 30–60 s. This is due to the higher growth rate of its crystals [5]. Therefore, the addition of setting retarders such as citrate, pyrophosphate, sulphate, or phytate is necessary for clinical use [6].

Brushite cements have a significantly better resorbability compared to hydroxyapatite [7]. However, their biodegradation is reduced in vivo through the conversion of brushite to hydroxyapatite and other poorly resorbable calcium phosphates [8]. This biodegradation is already observable at physiological pH values through passive resorption and hydrolysis [5], while the resorbability of hydroxyapatite only increases in acidic conditions [9]. Nonetheless, further advancements are needed to optimize their properties and overcome limitations such as inadequate initial mechanical strength [10,11].

To improve the performance of calcium phosphate cements, various mineral additives have been explored. In addition to fibre reinforcement [12], which is often non-degradable [13], dual-setting cements have been developed as a new modification of CPCs and other cements [14,15,16]. In these cements, the liquid phase of the cements is modified by water-soluble monomers, which polymerise during precipitation to form a hydrogel matrix [17,18]. In this context, silicate matrices based on tetraethyl orthosilicate (TEOS) have shown considerable promise. These materials possess unique characteristics that make them highly suitable for biomedical applications. Their composition allows the controlled release of bioactive ions, such as silica, calcium, and phosphate, which can stimulate cellular responses and promote osteogenesis [19,20]. Silicate matrices also offer improved mechanical stability, which addresses the brittleness issue associated with traditional calcium phosphate cements [21].

The mechanical properties of calcium phosphate cements are critical for load-bearing applications and successful integration with surrounding bone tissues. Silicate matrices have been shown to enhance the compressive strength, flexural strength, and fracture toughness of calcium phosphate cements [22,23]. These improvements are attributed to the formation of a durable silicate network, reinforcing the overall structure of cements [21].

The aim of this study was to improve the physical and biological performance of our dual-setting calcium phosphate cement. Therefore, 1,8-bis(triethoxysilyl)octane was used as a monomer in addition to TEOS to achieve greater crosslinking of the silicate matrix monomers to reach higher initial compressive strength and better cytocompatibility [24]. This technique of dual-setting cements was also used to modify brushite–baghdadite cements (BCB) already developed by No et al., who produced a composite cement of brushite and baghdadite to increase the biocompatibility of brushite cements [25].

## 2. Materials and Methods

### 2.1. Sample Fabrication

For β-TCP, sintering was performed by combining dicalcium phosphate (CaHPO_4_, Merck, Darmstadt, Germany) and calcium carbonate (CaCO_3_, Merck, Darmstadt, Germany) at a 2:1 molar ratio and subjected it to 1050 °C for 5 h. Subsequently, the sintered cake was finely ground using a planetary ball mill (Retsch PM400, Haan, Germany) for 1 h. A particle size d_50_ of 14.5 µm was obtained.

Baghdadite (Ca_3_ZrSi_2_O_9_) powder was synthesised by mixing zirconia (ZrO_2_, Sigma, Darmstadt, Germany), CaCO_3_ (Merck, Darmstadt, Germany), and silica (SiO_2_, Sigma, Darmstadt, Germany) in a ratio of 1:3.4:2.3 for 2 h in a ball mill (Retsch PM400, Haan, Germany). The powder mixture was sintered at 1400 °C for 3 h, followed by manual crushing and ball milling for 30 min. Finally, a particle size d_50_ of 2.7 µm was obtained.

Subsequently the synthesized powders underwent phase purity analysis via X-ray diffraction (XRD).

The powder phase of the unmodified brushite cement samples were prepared by mixing equimolar amounts of β-TCP and monocalcium phosphate anhydrous (MCPA, Ca(H_2_PO_4_)_2_, Budenheim, Germany), with a particle size d_50_ of 21.1 µm. For the cement samples modified with baghdadite (BCB), the β-TCP reactant was replaced with 20 wt% baghdadite. These recipes are shown in Table 1.

The liquid phase consisted of silicate matrix precursors and distilled water in a ratio of the alcoxy group to water of 2.25. Tetraethyl orthosilicate (TEOS, Sigma-Aldrich GmbH, Steinheim, Germany) was used alone in one batch and combined with a 30 mol% of 1,8-bis(triethoxysilyl)octane (TEOS-OC, Gelest, Morrisville, PA, USA) in another. Silica gel was prepared through the sol-gel reaction under acidic conditions adding 0.1 M HCl, creating an acid-catalysed sol. The paste was mixed with a powder-to-liquid ratio (PLR) of 2.0 g/mL. These recipes are based on a dissertation by I. Holzmeister from our research group [26]. These compositions are shown in Table 2. Also, the powder phase contained 1 wt% citric acid (C_6_H_8_O_7_, Sigma Aldrich, Steinheim, Germany), as processing without setting retarders was not possible.

The cement pastes were moulded using silicon forms and allowed to harden for 7 d at 37 °C and 100 % humidity.

### 2.2. Characterisation

To determine the initial setting time, a Gillmore needle test was carried out in a container at ≥90% humidity and 37 °C. The specimens used for compressive strength assessment were cuboids measuring 6 × 6 × 12 mm. Compressive strength tests were conducted using the universal testing machine Z010 (ZwickRoell, Ulm, Germany) with a crosshead speed of 1 mm/min. Eight samples were tested for each formulation.

X-ray diffraction (XRD) analysis was carried out using a D8 Advance with a DaVinci design diffractometer (Bruker AXS, Karlsruhe, Germany) to verify the phase compositions of the cements. The analysis involved measuring an angle range from 7° to 70° (2θ) with a step size of 0.0112° and integration time of 0.2 s, utilizing copper K_α_ radiation.

The specimens used for biological testing were prepared as discs with a diameter of 5 mm and a height of 2 mm for incubation with RAW 264.7 cells (ATCC no. TIB-71, Rockville, MD, USA) and as discs with a diameter of 15 mm and a height of 2 mm for incubation with MG-63 cells (ATCC no. CRL-1427, Rockville, MD, USA). The discs were then washed 10 times for 1 h in PBS. To monitor the pH development of the hardened specimens, the pH value of the PBS washing solution was measured hourly using an InoLab Level 1 pH meter (Xylem Analytics Germany Sales GmbH & Co. KG, WTW, Weilheim, Germany) until a physiological pH value was achieved.

### 2.3. Biological Testing

For biological testing, all scaffolds underwent γ-sterilization by BBF GmbH (Kernen, Germany) at a dose of 32.6 kGy.

The osteoblast-like MG-63 cell line was cultured in Dulbecco’s Modified Eagle’s Medium (DMEM, Gibco, Carlsbad, CA, USA, Cat. No.: 31966-021), supplemented with 10 % foetal calf serum (FCS, Gibco, Carlsbad, CA, USA, Cat. No.: 10270-106) and 1 % penicillin-streptomycin (Gibco, Carlsbad, CA, USA, Cat. No.: 15140-122). The specimens (*n* = 4) were carefully placed in 24-well plates using sterile forceps. To allow the specimens to set, they were incubated for 24 h with DMEM and then seeded with 5 × 10^4^ cells/well. Cell counting was performed using a CASY 1 cell analyser (Schärfe System, Reutlingen, Germany). The plates were incubated at 37 °C and 5 % CO_2_. The cell activity and cell number were analysed on day 2, 4, 7, and 9. For analysis, samples were incubated for 30 min with a 1:10 dilution of WST-1 reagent. Cell activity was then measured in duplicates using a microplate reader (Tecan Spark^®^ 20M, Tecan, Maennedorf, Switzerland). The cell number was determined again using the Casy cell counter after detaching the cells from the surface of the samples through a 12 min incubation with Accutase (Sigma Aldrich, Steinheim, Germany, A6964).

Furthermore, to assess the biocompatibility on osteoclastic cells, murine Raw 264.7 cells were cultured in Dulbecco’s Modified Eagle’s Medium (DMEM, Gibco, Carlsbad, CA, USA, Cat. No.: 31966-021), supplemented with 10 % foetal calf serum (FCS, Gibco, Carlsbad, CA, USA, Cat. No.: 10270-106) and 1 % penicillin-streptomycin (Gibco, Carlsbad, CA, USA, Cat. No.: 15140-122). Cells up to passage 12 were utilized. The specimens (*n* = 4 for quantitative TRAP and DNA testing; *n* = 2 for TRAP staining and SEM) were placed in 96-well plates and seeded with 2 × 10^4^ cells/cm^2^. Cell counting was also performed using the CASY cell counter. Differentiation was achieved by adding 50 ng/mL RANKL (R&D systems, Minneapolis, MN, USA, Cat. No.: 462-TEC). The medium containing 50 ng/mL RANKL was renewed every 48 to 72 h. Each sample was measured twice, and the quantifiable data were calculated as the mean value along with its standard deviation. Additionally, the statistical analysis was performed using a *t*-test.

Tartrate-resistant acid phosphatase (TRAP) serves as a specific marker for osteoclastic differentiation. To establish intracellular TRAP activity, RAW 264.7 cells were lysed. Then, the cement surfaces were rinsed repeatedly with PBS, and the specimens (*n* = 4) were transferred to a new plate. Subsequently, they were incubated in 500 µL of 1 % Triton X-100 (Merck, Darmstadt, Germany) on ice for 60 min, and the lysates were preserved by freezing them at −80 °C. The quantification of TRAP was performed by assessing the conversion of p-nitrophenyl phosphate (pNPP, Sigma-Aldrich, Steinheim, Germany) to p-nitrophenol (pNP). To 50 µL of lysate, 150 µL of substrate solution (100 mM sodium acetate, 50 mM disodium tartrate dehydrate, and 7.6 mM pNPP) was added and incubated for 60 min at 37 °C and 5 % CO_2_. The enzyme reaction was halted by adding 50 µL of 3 M NaOH, and the absorbance was measured in duplicates using the Tecan spectrometer at a wavelength of 405 nm.

To analyse the proliferation of RAW 264.7 cells on the cement surface (*n* = 4), the DNA concentration was detected. For this purpose, 20 µL of lysate were added to 180 µL of the PicoGreen solution (1:800 PicoGreen reagent dilution with TE buffer; Invitrogen, Karlsruhe, Germany, Cat. P7589) in black 96-well plates and measured in duplicates using the Tecan plate reader (extinction 485 nm, emission 535 nm).

Each sample was subjected to two measurements, and the quantifiable data were determined as the mean value along with its standard deviation. Furthermore, a *t*-test was performed for statistical analysis.

To visualize the differentiation of cell clusters into osteoclasts, TRAP staining was conducted using a commercial TRAP staining kit (Sigma, Steinheim, Germany, Cat No. 387). RAW 264.7 cells were incubated on the samples with 100 µL of fixing solution for 30 s, rinsed twice with deionized water, and subsequently incubated with 100 µL of staining solution for 60 min. After rinsing the samples again, they were dried and analysed using a stereomicroscope (Discovery V20, Zeiss, Germany).

For the examination of cell structure, scanning electron microscopy (SEM) was performed. RAW 264.7 cells were fixed with 6 % glutaraldehyde for 15 min at 0 °C. The samples were then dehydrated using an ascending series of ethanol (30%, 50%, 70%, 90%, and 100%) for 30 min with the latter step repeated five times. Afterwards, the samples were dried with hexamethyldisilazane (HMDS) for 15 min. A platinum layer of 4 nm was sputtered onto the specimens with the ACE600 sputter coater (Leica, Wetzlar, Germany), and the scanning was performed using Crossbeam 340 (Zeiss, Oberkochen, Germany) at an acceleration voltage of 5.0 kV and a 1000-fold magnification.

The solubility of the cements was assessed by collecting the medium of differentiated RAW 264.7 cells incubated on the surfaces of the cement specimens. In addition, the medium of cements immersed without cells was also collected as a reference. These samples (*n* = 4) were collected on day 3, 6, 8, 10, 13, and 15 during the biocompatibility experiments and stored at −80 °C. Quantification of ion concentrations (Ca, Si, and P) was carried out using inductively coupled plasma mass spectrometry (ICP-MS iCAP RQ, Thermo Fisher Scientific, Waltham, MA, USA, Cat. No. BRE731416) against standard solutions of Ca(NO_2_)_2_ (Merck, Darmstadt, Germany, Cat. No. 170308), SiO_2_ (Merck, Darmstadt, Germany, Cat. No. 170365), and H_3_PO_4_ (Merck, Darmstadt, Germany, Cat. No. 170340).

Passive resorption was measured by analysing the reference eluates, while total resorption was measured using the eluates under the influence of RAW 264.7. The active resorption, which represents the influence of RAW 264.7 independent of passive resorption, was calculated by determining the deviation between total and passive resorption.

## 3. Results

### 3.1. Phase Analysis

**Figure 1:**
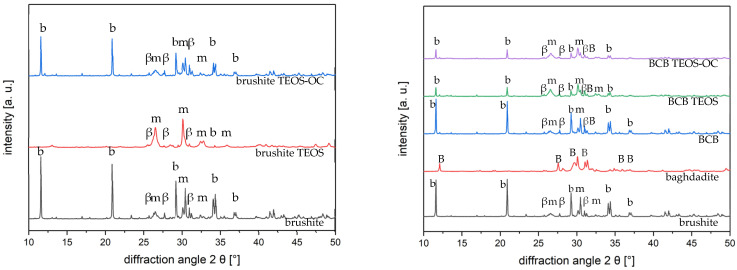
Diffraction pattern of the phase composition of brushite and BCB cements as reference and as dual-setting cements (TEOS and TEOS-OC) (b brushite ICDD #09-0077, m monetite ICDD #09-0080, β β-TCP ICDD #09-0169, B baghdadite ICDD #54-0710). The patterns were calculated using TOPAS 4.2 software (Bruker AXS, Karlsruhe, Germany).

The phase analysis of the cements was conducted after 7 d of hardening in 37 °C water, and an additional drying period with X-ray diffraction (XRD) is shown in Figure 1. In brushite and BCB cements, the three phases, brushite, monetite, and β-TCP, were detected. The highest peaks were corresponding to brushite and monetite (due to the drying phase, high peaks in monetite were observed), whereas β-TCP was less represented in our analysis. In BCB cements, only small peaks of the diffraction pattern could be identified for baghdadite. In addition, Table 3 shows the Rietveld analysis of the XRD analysis.

### 3.2. pH Value

**Figure 2:**
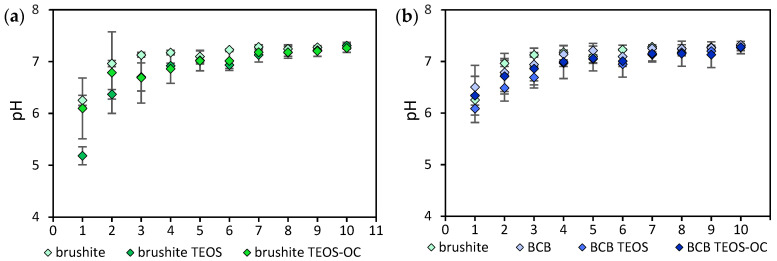
pH profile of the washing solution (*n* = 3) in which the cements of brushite (**a**) with TEOS or TEOS-OC; and BCB (**b**) with TEOS or TEOS-OC were placed. Each washing cycle took 1 h. The error bars represent the standard deviation.

The pH profiles of the washing solution of calcium phosphate cements with different silicate matrices and substitution with baghdadite are illustrated in Figure 2. The starting pH value was low in every composition due to its formation under acidic conditions.

Among the pure brushite cements, brushite TEOS with 5.18 ± 0.17 showed the lowest pH value up to the third washing cycle. From the seventh washing cycle onwards, however, physiological pH values were obtained for this cement series.

For the BCB cements, comparable results were obtained for the pH values. The lowest initial pH value of 6.09 ± 0.27 was also found for the dual-curing BCB TEOS cement.

Here, too, physiological pH values were exhibited after seven washing cycles with BCB 7.25 ± 0.07, BCB TEOS 7.13 ± 0.14, BCB TEOS-OC 7.15 ± 0.14.

Contrary to that, the washing solutions of pure baghdadite cements had more alkaline pH values. After initial pH values of 8.85 ± 0.58, these cements only showed an approximation to a physiological pH environment up to 8.01 ± 0.23.

### 3.3. Compressive Strength

**Figure 3:**
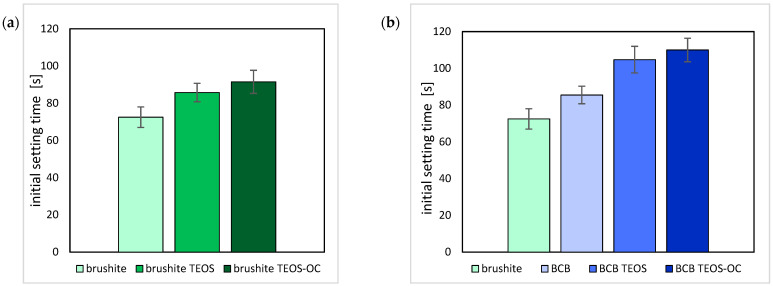
Initial setting times as determined by the Gillmore needle test of brushite (**a**) with TEOS or TEOS-OC; and BCB (**b**) with TEOS or TEOS-OC. The error bars represent the standard deviation (*n* = 8).

Firstly, the initial setting times of the cements were analysed using the Gillmore needle test to investigate the hardening of the cement (Figure 3). The cements showed a very short setting time despite the addition of citric acid. This was slightly extended by the addition of the silicate matrix.

**Figure 4:**
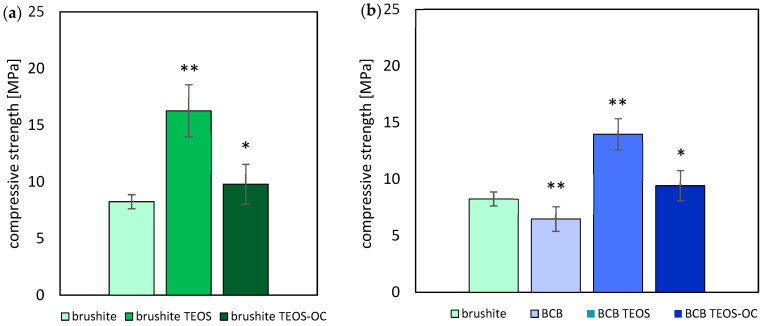
Compressive strength of cuboid brushite (**a**) and BCB (**b**) specimens with different formulations of silicate matrices ∗ = *p* < 0.05; ∗∗ = *p* < 0.005; the error bars represent the standard deviation (*n* = 8).

Figure 4 compares the compressive strength of the different formulations of the dual-setting cements based on brushite and BCB. Regarding the brushite cements, the addition of silicate matrices for brushite TEOS resulted in the highest compressive strength with 16.27 ± 2.31 MPa. When prepared on the basis of two alkoxysilanes, the compressive strength of the dual-setting cement brushite TEOS-OC increased with 9.80 ± 1.77 MPa to the level of the brushite cement with 8.25 ± 0.62 MPa.

Comparable results were obtained for the composite BCB cements of brushite and baghdadite. While the addition of baghdadite to the brushite cements led to a decrease in compressive strength, BCB TEOS achieved the highest compressive values 13.97 ± 1.38 MPa. BCB TEOS-OC, however, also showed an increase in the values with 9.42 ± 1.35 MPa to the level of the brushite cement, whereby these values were above those of BCB.

### 3.4. Osteoblastic-Like Cells

**Figure 5:**
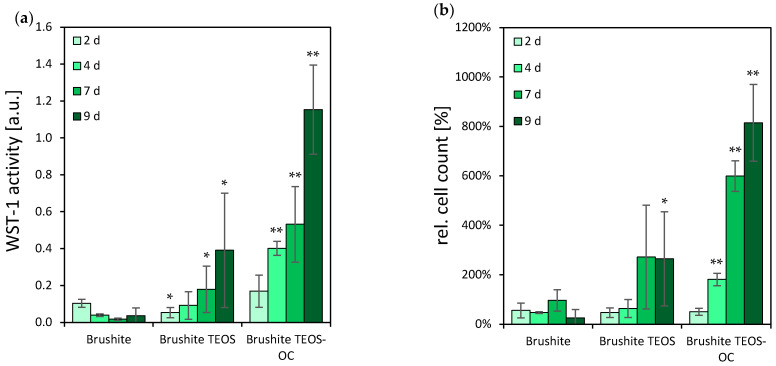
Cell activity (**a**) and cell number (100% at 50,000 cells) (**b**) of MG-63 on brushite specimens with regard to different compositions of silicate matrices. WST-1 activity and cell proliferation in comparison to the brushite reference ∗ = *p* < 0.05; ∗∗ = *p* < 0.005; the error bars represent the standard deviation (*n* = 4).

Throughout the entire 9 d testing period, the cement composition of brushite TEOS-OC showed the highest WST-1 activity of the MG-63 osteoblastic-like cell-line when exposed to the surfaces of the tested dual-setting brushite cements. In addition, for brushite TEOS, a significantly higher level of activity than the control could be detected after 9 d of incubation. The latter, however, exhibits a comparable relative cell count (the initial added cell number of 50,000 cells per well was set at 100%) to the control as shown in Figure 5, whereas brushite TEOS-OC had, significantly, the highest cell counts from day 4.

**Figure 6:**
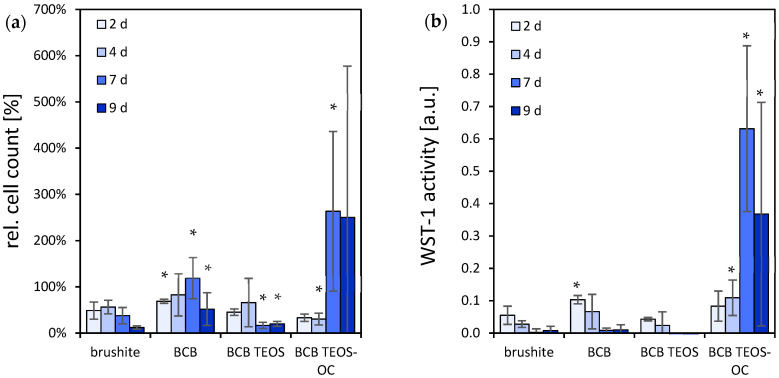
Cell activity (**a**) and cell number (100% at 50,000 cells) (**b**) of MG-63 on brushite and BCB specimens with regard to different compositions of silicate matrices. WST-1 activity and cell proliferation in comparison to the brushite reference ∗ = *p* < 0.05; the error bars represent the standard deviation (*n* = 4).

Similarly, MG-63 cells on BCB samples (Figure 6) showed a greater increase when the dual-setting cements were created with two alkoxysilanes. The cements composed only with TEOS, on the other hand, exhibited significantly lower cell activity with comparable values to the control. The cells on the latter showed a significant decrease in cell activity over the testing period. Likewise, the highest cell count of the BCB cements was found for BCB TEOS-OC after 7 d. On the other specimens, however, a slight decrease in cell count was registered to less than half the original cell count.

### 3.5. TRAP Activity Quantitative

**Figure 7:**
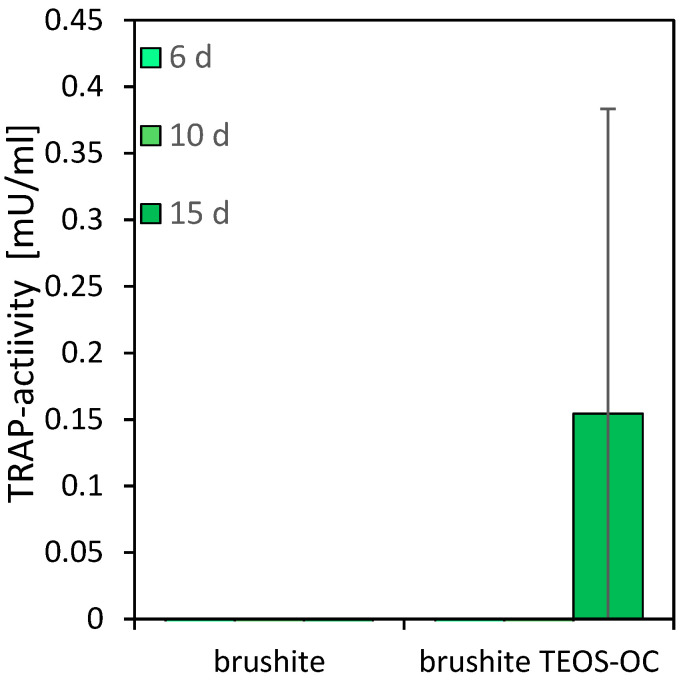
TRAP activity of RAW 264.7 cells incubated with 50 ng/mL RANKL on brushite cements with and without silicate matrices over 15 d. TRAP activity in comparison to the brushite reference; the error bars represent the standard deviation (*n* = 4).

**Figure 8:**
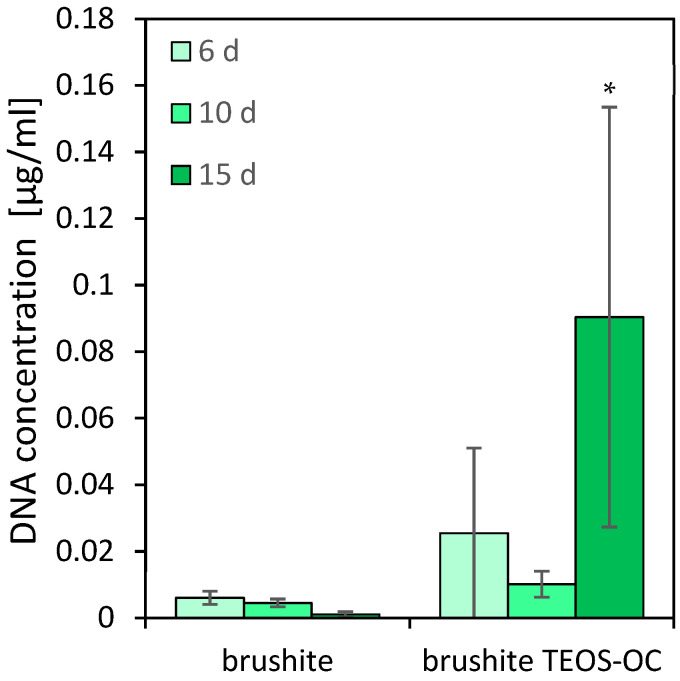
DNA concentration of RAW 264.7 cells incubated with 50 ng/mL RANKL on brushite cements with and without silicate matrices over 15 d. DNA-concentration in comparison to the brushite reference ∗ = *p* < 0.05; the error bars represent the standard deviation (*n* = 4).

A well-established method for investigating the cytocompatibility of cements with osteoclastic cells is to culture RAW 264.7 cells on their surfaces. This macrophage cell line was differentiated by adding 50 ng/mL RANKL to the culture medium. To quantify the differentiation and activity of the RAW cells, the activity of the specific enzyme TRAP and the DNA concentration were measured. The results are shown in Figure 7 and Figure 8.

While no activity of RAW 264.7 osteoclasts could be detected on the surface of the control specimens, the dual-setting brushite cement showed an increase after 15 d of differentiation with RANKL. Similarly, the DNA concentration on the control cement decreased over the testing period. Additionally, the cells on the dual-setting cement showed an increase in DNA concentration with significant higher values after 15 d of cultivation.

### 3.6. SEM

**Figure 9:**
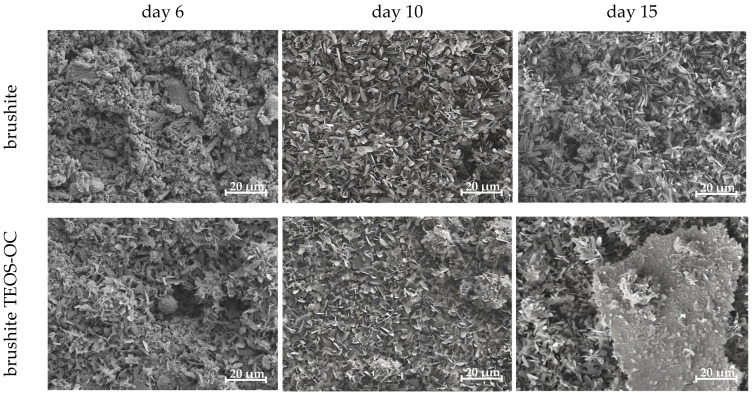
SEM images of RAW 264.7 differentiated with RANKL on the surface of the various calcium phosphate cements with and without TEOS-OC at day 6, 10 and 15. The images were made at 1000× magnification and given with a scale of 20 μm.

The verification of RAW 264.7 cell differentiation into polynuclear osteoclastic cells was performed by scanning electron microscopy (SEM) (Figure 9) analysis of the cement surfaces.

When examining the brushite control specimens produced with citric acid, SEM analysis initially revealed the presence of mononuclear cells in addition to the cement structure, whereas after 10 d, only cell detritus was apparent. The dual-setting cement brushite TEOS-OC, on the other hand, showed clear signs of cluster formation at the edges of the test specimens after 15 d in SEM, where the initially round mononuclear cells on the surface of the dual-setting cement first showed a flattening and finally reached a diameter of >40 µm after cultivation with RANKL.

### 3.7. Solubility

**Figure 10:**
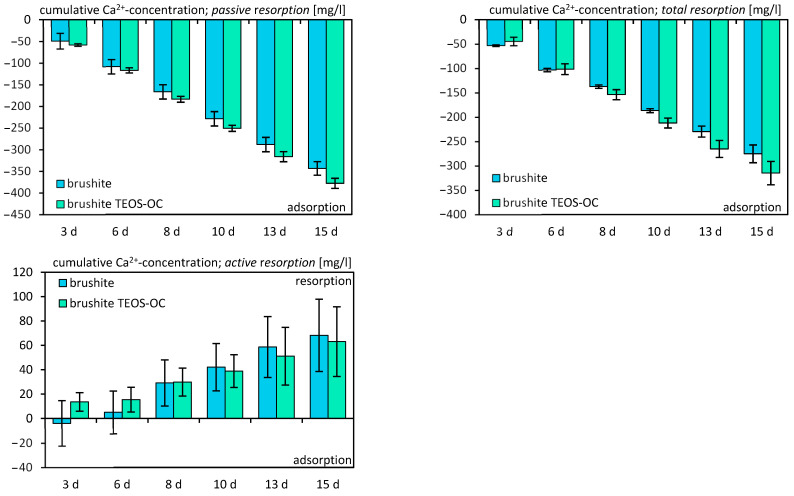
Cumulative Ca^2+^ ion concentration due to their release by passive, total, and active resorption of calcium phosphate cements for 15 d. The specimens were incubated without cells for passive resorption and with cells for total resorption, and the difference between those two was formed for active resorption. The error bars represent the standard deviation (*n* = 4).

**Figure 11:**
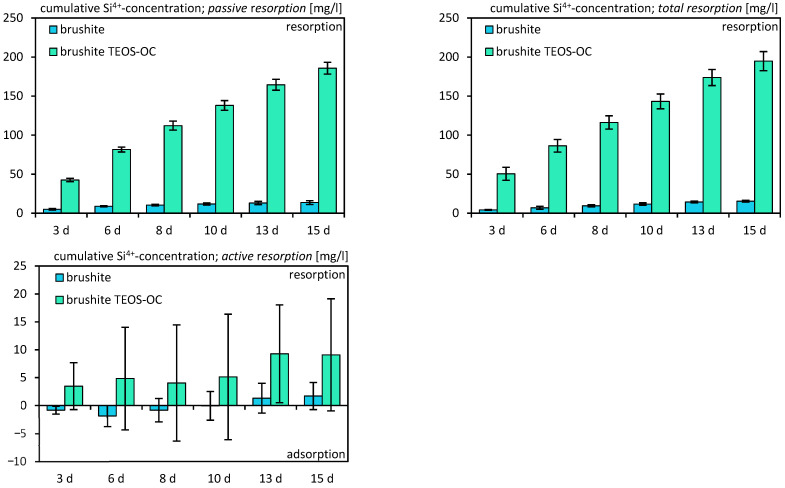
Cumulative Si^4+^ ion concentration due to their release by passive, total, and active resorption of calcium phosphate cements for 15 d. The specimens were incubated without cells for passive resorption and with cells for total resorption, and the difference between those two was formed for active resorption. The error bars represent the standard deviation (*n* = 4).

**Figure 12:**
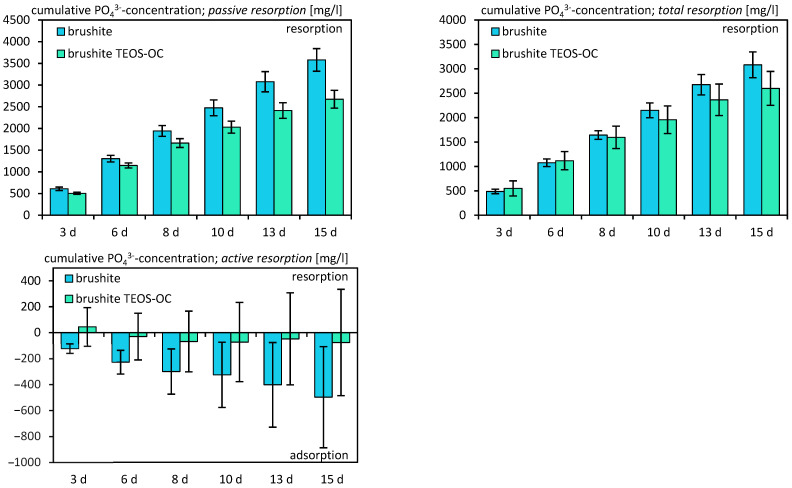
Cumulative PO_4_^3−^ ion concentration due to their release by passive, total, and active resorption of calcium phosphate cements for 15 d. The specimens were incubated without cells for passive resorption and with cells for total resorption, and the difference between those two was formed for active resorption. The error bars represent the standard deviation (*n* = 4).

The evaluation of cement solubility involved the preservation of the utilized culture medium. This assessment was conducted in two scenarios: firstly, without cells to determine passive solubility, and secondly, with differentiated RAW 264.7 cells to measure total solubility. Subsequently, the medium underwent analysis using inductively coupled plasma (ICP) techniques. The dual-setting cement and the control cement both exhibited calcium adsorption (Figure 10) when compared to the fresh cell medium, regardless of the presence or absence of RAW 264.7 cells. However, the active absorption of calcium showed only marginal positive values. As expected, the release of silicon (Figure 11) was significantly higher for the dual-setting cements than for the reference cements. Also, an increase could be detected by the cultivation of RAW 264.7. In contrast, both cements demonstrated a notable release of phosphate (Figure 12), with the reference cement exhibiting significantly higher values compared to the other cement.

**Figure 13:**
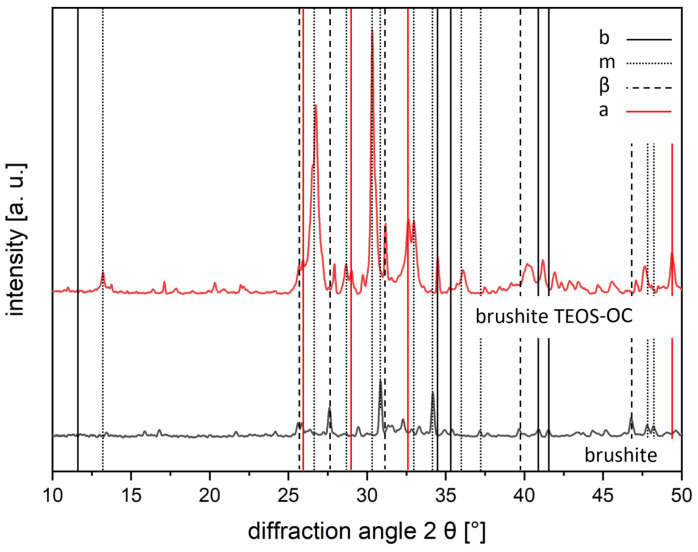
Diffraction pattern of the phase composition of brushite cement as reference and dual-setting cement TEOS-OC (b brushite, m monetite, β β-TCP, a hydroxyapatite). The patterns were calculated using TOPAS 4.2 software (Bruker AXS, Karlsruhe, Germany).

As calcium resorption of the brushite cements was more likely to be expected, another XRD analysis of the cements was carried out as shown in Figure 13 to analyse the mineral phases of the cements again. This revealed an increase in hydroxyapatite on the surface of the cements, which was predominant in the dual-setting cement.

## 4. Discussion

The objective of this study was to improve our cement formulation with dual-setting capabilities. To achieve this, a brushite-forming calcium phosphate cement (CPC) was combined with a TEOS-based silica gel, and the silica matrix was modified with 1,8-bis(triethoxysilyl)octane. Unlike previous works that utilized either silica-modified CPC [27,28] or calcium-phosphate-modified silica gels [29,30], our approach involved the simultaneous setting of both of these inorganic components, resulting in the formation of two interconnected matrices. This distinctive feature sets our material apart by eliminating the use of an unreactive filler component within the matrix-forming process.

The basis of the cement developed in this work was established by our research group in 2015 [21]. In this article, a successful synchronization of the setting reaction of the cement with the silica condensation reaction was achieved, ensuring solidification occurred within a similar timeframe for both reactions. In this system, brushite sets through a precipitation reaction involving the dissolution of β-TCP under acidic conditions. On the other hand, the formation of silica matrices follows a sol-gel process, which involves the hydrolysis of monomers under acidic conditions, followed by condensation to create a silicate network [31]. To stimulate the condensation of TEOS chains and to create further crosslinks, another monomer 1,8-bis(triethoxysilyl)octane was added to the TEOS monomers in our approach.

Furthermore, the composite cement of brushite and baghdadite developed by No et al. was modified into a dual-setting cement with a silicate matrix [25]. This approach was intended to improve the condensation reaction of the silicate network, which occurs at higher pH values than the hydrolysis of the monomers [32]. The latter could be observed in each of the modifications as a slight increase in pH for all compositions (Figure 2).

In our study, the dual-setting cement containing TEOS exhibited compressive strength values of up to 16.27 ± 2.31 MPa (PLR = 2 g/mL). These values are almost twice as high as the reference cement and up to four times higher than comparable CPC cements (Figure 3). On the other hand, the addition of 1,8-bis(triethoxysilyl)octane could only improve the compressive strength slightly in comparison to the brushite reference. The compressive strength of the BCB reference cement was relatively low due to the inherent fragility of baghdadite. However, notable improvements in compressive strength were observed in the BCB cement incorporating TEOS and 1,8-bis(triethoxysilyl)octane. We attributed these enhancements to the higher pH value and the resulting improved condensation reaction [32].

Geffers et al. found a significant increase in cell activity and number on the CPC–silica gel composites during cultivation with osteoblastic cells, in addition to the improved compressive strength [21]. In this study, only a slight increase of biocompatibility of osteoblastic cells on the surface of dual-setting cements based on TEOS was observed. However, the dual-setting cements produced with a combination of TEOS and 1,8-bis(triethoxysilyl)octane showed, over the whole observation period, a significant increase in cell activity, more than 10-folds higher compared to all brushite reference values, and an 8-fold increase in cell number after 15 d of cultivation. This difference in our results compared to Geffers et al. could be linked to the use of citric acid as a setting retardant, which was used in all our test specimens, whereas Geffers et al. used citric acid only in their reference. These results support those of Jamshidi et al., who found that adhesion of osteoblastic cells was reduced by citric acid through the formation of diverse citric complexes [33].

As citrate serves as an organic catalyst in the sol-gel reaction, its addition leads to the crosslinking of carbon atoms between the citrate and the silicate matrix [34]. This crosslinking process is suggested by the authors to reduce the formation of amorphous dicalcium citrate phases in brushite cements when further alkoxysilanes, like 1,8-bis(triethoxysilyl)octane, are added. Moreover, the cytocompatibility increased corresponding to the number of silyl groups present in the precursors.

No et al., who investigated compound cements of brushite and baghdadite, found that an extract of the BCB cements leads to an improvement in cell activity of primary human osteoblasts, compared to brushite cements [25]. In contrast to that, the osteoblastic cells were cultivated directly on the surface of the cement specimens. This reflects the physiological requirements of implantable materials better, as surface properties are also investigated [35]. No et al. attributed the improved biocompatibility to the reduced release of phosphate from the BCB cements [25]. On the other hand, only a slight increase of the biocompatibility on the surface of latter cements could be observed in our study, while the cells on the surface of the dual-setting BCB TEOS and 1,8-bis(triethoxysilyl)octane showed a significant increase in cell activity. As with the brushite cements, we also attribute the lower biocompatibility of the BCB cements to the use of citrate. However, the difference in results between No et al. and this study may also be attributed to the cultivation of cells on the surface of the cements.

In this study, the RAW 264.7 mesenchymal cell line was utilized to examine the biocompatibility of the cements. These established cells can be differentiated into osteoclast-like cells using RANKL [36,37]. To avoid bias towards capturing only cells with ≤5 nuclei, which exhibit limited resorption abilities, cell differentiation on the specimens was also assessed through scanning electron microscopy [38,39].

The biocompatibility of dual-setting brushite cements with a silica matrix has not been investigated using osteoclastic cells yet. Heinemann et al. conducted experiments using silicate-collagen xerogels based on TEOS, supplemented with varying concentrations of hydroxyapatite [30]. They observed an increase in DNA concentration and TRAP activity of human osteoclasts when calcium phosphate was added. Also, Vahabzadeh et al. observed increased differentiation of RAW 264.7 cells into osteoclasts on brushite cements doped with silicon [40]. Surprisingly, the highest TRAP activity was observed with the lowest silicon doping, suggesting an inhibitory effect. Furthermore, in vivo studies have shown that doped cements lead to an increase in bone formation without any phase changes when compared to non-doped brushite cements. In this study, the dual-setting cements exhibited higher DNA concentrations compared to the brushite control. The brushite control showed a significant increase after 15 days, which is consistent with the findings of Heinemann et al. [30]. The low DNA concentration and TRAP activity on the control cement align with the results of Meininger et al., who observed reduced adhesion of RAW osteoclasts on brushite specimens prepared with citrate as a retarder [6].

An increase in TRAP activity was observed towards the end of the observation period on the dual-setting brushite cements, indicating the initiation of differentiation into resorbing osteoclasts on these cements. This was also reflected by the TRAP staining and SEM where initial cell clusters on the dual-setting cements were recorded. These findings were attributed by the authors as well to the reduction of the formation of amorphous dicalcium citrate phases. Although the investigated cements are not doped with silicate, an examination of the weight proportion of silicate should be conducted to assess the maximum effect.

The analysis of ion concentrations revealed increasing adsorption of calcium on both brushite cements, with or without RAW 264.7 cells. The resorption of phosphate also increased over time but was not influenced by the cultivation of RAW 264.7 cells. The release of phosphates is associated with the adsorption of calcium on the specimens, resulting in the transformation of the cements into more complex calcium phosphates with a high calcium-to-phosphate ratio like hydroxyapatite or amorphous minerals, which is characteristic of brushite cements [41,42,43]. To confirm this, we were able to detect an increase in hydroxyapatite and amorphous minerals on the surface of the specimens in further XRD investigations. The comparable results of Schamel et al. support our findings [44]. However, in the current study, a correlation between the conversion of brushite to CDHA and the ion concentration was also shown.

## 5. Conclusions

While there was an increase in physical properties for the dual-curing cements with TEOS, those of the cements with an additional precursor were in the range of the reference. However, the biocompatibility of the cements increased with the number of alkoxysilyl groups, which can be attributed to the higher crosslinking of the silicate matrix and the resulting smaller pore size. In addition, the setting retarder citrate, which led to a reduction in biocompatibility in brushite cements, also reacted with the silicate matrix in the sol-gel reaction. As a reduction in biocompatibility by citrate due to loss of cell adhesion is known from recent research, the increase in biocompatibility in our study can be attributed to its interaction with the silicate matrix. It is suggested by us that besides the use of dual-setting cements, citrate should be replaced by other setting retarders such as phytic acid, which has already shown good results in complementary studies in cell trials [6].

## Figures and Tables

**Table 1:** Recipes of the powder phase for the production of cements.

Sample Labelling	Baghdadite [g]	β-TCP [g]	MCPA [g]
brushite	0	11.04	8.31
brushite TEOS	0	11.04	8.31
brushite TEOS-OC	0	11.04	8.31
BCB	2.24	8.82	8.31
BCB TEOS	2.24	8.82	8.31
BCB TEOS-OC	2.24	8.82	8.31

**Table 2:** Recipes of the liquid phase for the production of cements, which were mixed with powders in a PLR of 2.0 g/mL.

Sample Labelling	H_2_O [mL]	TEOS [mL]	1,8-Bis(triethoxysilyl)octane [mL]
brushite	5.11	0	0
brushite TEOS	5.11	7	0
brushite TEOS-OC	8.31	7	6.39
BCB	5.11	0	0
BCB TEOS	5.11	7	0
BCB TEOS-OC	8.31	7	6.39

**Table 3:** Rietveld analysis of the XRD analysis.

Sample	Brushite [%]	β-TCP [%]	Monetite [%]	Baghdadite [%]
brushite	26.7	29.5	43.8	0
brushite TEOS	11.1	2.9	85.0	0
brushite TEOS-OC	32.5	23.0	44.4	0
BCB	46.3	9.5	35.6	8.6
BCB TEOS	6.2	8.9	71.2	6.2
BCB TEOS-OC	15.6	5.5	69.8	9.4

## Data Availability

The data presented in this study are available on request from the corresponding author. The data are not publicly available because ongoing currently unpublished studies are based on them.
